# Biophysical Consequences for Exposure of Model Cell Membranes to Perfluoroalkyl Substances

**DOI:** 10.1021/acs.jpcb.5c02472

**Published:** 2025-07-29

**Authors:** Joseph Fosella, Jasmin Ceja-Vega, Amani Rabadi, Micaela Panella, Jessica Said, Wilber Perla, Christopher Poust, Mary Herrera, Sunghee Lee

**Affiliations:** Department of Chemistry and Biochemistry, Iona University, 715 North Avenue, New Rochelle, New York 10801, United States

## Abstract

There has been a rising concern about negative impacts of per- and polyfluoroalkyl substances (PFAS) on human and environmental health, given the environmental persistence and bioaccumulation potential of PFAS. In this study, two exemplary PFAS, a long-chain perfluorooctanoic acid (PFOA) and a short-chain alternative perfluorobutanesulfonic acid (PFBS), are investigated to assess their potential to modify bilayers of model membranes formed from 1,2-dioleoyl-*sn*-glycero-3-phosphocholine (DOPC). A comprehensive suite of experimental techniques, including water permeability assays, thermal phase behavior analysis (DSC), vibrational spectroscopy (Raman and ATR-FTIR), and evaluations of interfacial properties, reveals concentration-dependent perturbations to DOPC membranes. Water permeability measurements reveal biphasic characteristics in PFAS-membrane interactions, corroborated by phase separation observed via DSC. PFOA and PFBS exhibit distinct impacts on membrane properties, reflecting a sensitivity to PFAS molecular structures. Higher membrane/water partition coefficients for PFOA underscore the role of hydrophobic effect in long- versus short-chain PFAS interactions. PFOA demonstrates a more pronounced effect than PFBS at lower concentrations, but they both exhibit similar impacts on DOPC membranes at higher levels. Notably, PFBS’s significant membrane modifications at high concentrations challenge the assumption that shorter-chain PFAS alternatives are inherently safer. These findings highlight the complex nature of PFAS-membrane interactions and emphasize the importance of molecular structure in assessing environmental and health impacts.

## Graphical abstract

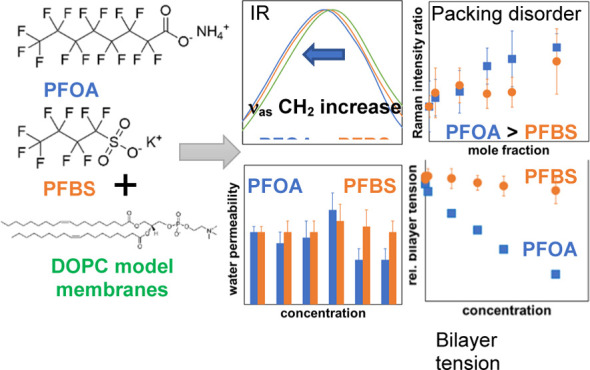

## Introduction

Per- and polyfluoroalkyl substances (PFAS) have been extensively used in various industrial applications and consumer products since the 1940s, primarily due to the unique properties of fluorocompounds,^1^ such as their exceptional thermal and chemical inertness. PFAS, defined as compounds in which one carbon is fully fluorinated and an adjacent carbon is at least partially fluorinated, comprise a family of nearly 15,000 distinct molecules.^2,3^ These substances are utilized in over 200 applications across a wide range of sectors, including industrial processes and consumer products such as food packaging and textiles.^4^ There is growing evidence and concern that PFAS, both in their original form and as degradation byproducts, adversely affect human and environmental health due to their widespread distribution and prolonged persistence in the environment.^5,6^ PFAS are known to accumulate in various human tissues^7^ and demonstrate significant interactions with key biological components, including transport proteins, nuclear receptors, and cellular membranes.^8^ The bioaccumulation of PFAS poses a broad range of adverse health risks, including inflammation, neurotoxicity, reproductive toxicity, cardiovascular toxicity, and various cancers, among others.^9–11^

As amphiphilic substances with high lipophilicity, some oxoanionic PFAS readily incorporate into cell membranes and alter their properties, inherently implying potential biotoxicological risks.^12^ These compounds exhibit diverse structures, with functional groups such as carboxylates and sulfonates as headgroups, and varying fluorocarbon chain lengths as tailgroups. Research has demonstrated that PFAS with different headgroups and carbon chain lengths show varying degrees of partitioning into lipid assemblies, indicating differing levels of bioaccumulation and membrane interactions based on their specific chemical characteristics. Generally, PFAS with sulfonate groups have a higher propensity to penetrate cells compared to carboxylate counterparts of the same chain length.^13^ Furthermore, within the same class, PFAS exhibit increased cellular uptake with longer chain lengths.^13^ For instance, interactions of perfluorocarboxylic acids with lipid bilayers intensify with longer perfluorocarbon chains, correlating with increased hydrophobicity.^14^ Among the various PFAS compounds, perfluorooctanoic acid (PFOA) and perfluorooctanesulfonic acid (PFOS) have been the most extensively studied regarding their biological effects and potential impacts on human health. In response to concerns about these long-chain PFAS, shorter-chain alternatives such perfluorobutanesulfonic acid (PFBS) are increasingly produced, aiming to enhance elimination and reduce bioaccumulation. However, the toxicological effects of these short-chain PFAS on human health and the environment remain inadequately understood.^15,16^

Zwitterionic phospholipid bilayers are frequently employed as model systems to represent eukaryotic cell membranes. While there is a growing body of evidence regarding the interactions between PFAS and zwitterionic lipid membranes, significant controversies remain concerning the nature and extent of membrane perturbation. Some studies suggest that the incorporation of PFAS into zwitterionic lipid bilayers will enhance lipid order, stiffen the lipid bilayer, increase bending rigidity, and stabilize liposomes against nanoparticle-induced leakage—effects akin to those observed with cholesterol.^17–19^ Conversely, other studies indicate a destabilizing effect, where PFAS can significantly disturb membrane properties. These perturbations include increasing the gauche conformation and mobility of lipid alkyl chains, leading to alkyl chain disordering and structural changes, and causing increased fluidity and disruption of the packing within the bilayer.^20–23^ Therefore, additional clarification is needed to gain a deeper understanding of how PFAS interact with cell membranes.

The structural and functional integrity of lipid bilayers is crucial for maintaining cellular homeostasis, as these bilayers provide the essential environment for membrane-associated processes. Any disturbance to the lipid organization can significantly alter the membrane’s physical properties, potentially affecting molecular transport, signal transduction, and the activity of membrane-embedded biomolecules, thereby disrupting overall cellular function.^24,25^ Model lipid membranes provide a simplified yet powerful approach to unravel the complexities of lipid-mediated interactions in biological membranes, offering crucial insights into the fundamental mechanisms that govern membrane dynamics and their interactions with external compounds.^26^ We utilized 1,2-dioleoyl-*sn*-glycero-3-phosphocholine (DOPC) to form a model membrane, due to its fluid phase at physiological temperatures, well-characterized physical properties, and ability to mimic the fluidity and thickness of many biological membranes.^27,28^ The literature indicates that PFAS structural features, such as headgroup identity and chain length, play important roles in determining the cellular distribution and toxicity of PFAS.^13^ In this study, we selected PFOA and PFBS, which differ in both headgroup and chain length. The primary molecular distinction between the two compounds lies in their structural composition: PFOA features an eight-carbon perfluorinated chain with a carboxyl functional group, whereas PFBS possesses a shorter four-carbon chain with a sulfonate group (Figure 1). By directly comparing PFOA and PFBS, we aim to elucidate how these specific differences influence lipid membrane properties. This study serves as an investigation into the combined effects of functional group and chain length on bilayer behavior. Our investigation employed a comprehensive suite of experimental techniques to assess biophysical properties, including water permeability assays, thermal phase behavior analysis, and vibrational spectroscopy (Raman and ATR-IR), as well as evaluations of interfacial properties. The use of multiple biophysical techniques provides a more complete picture of membrane behavior, recognizing that different methods may not always yield consistent trends since each method probes distinct molecular or structural features.^29^

**Figure 1:**
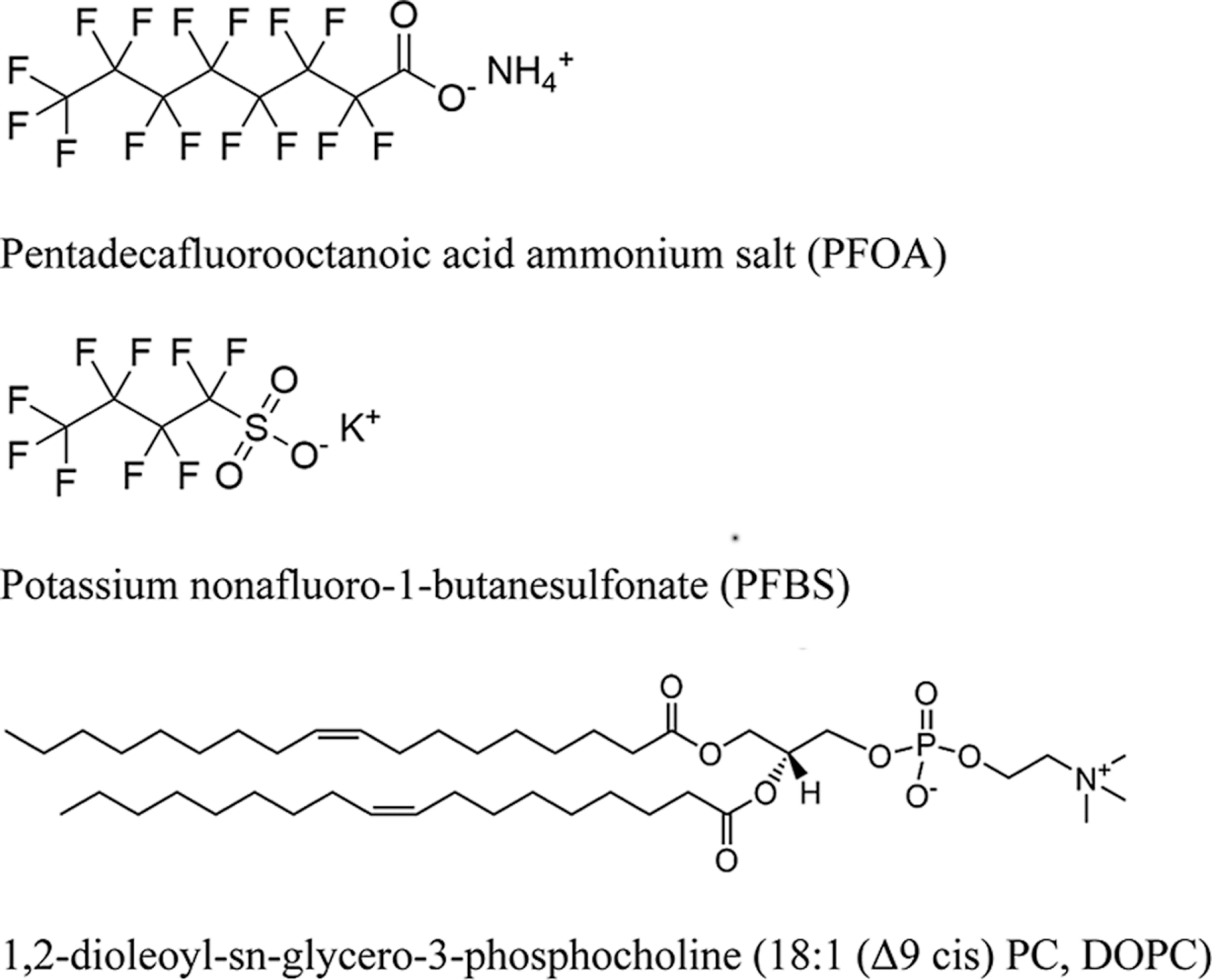
Molecular structures of PFOA, PFBS, and DOPC used in this study.

## Materials and Methods

### Sample Preparations

The lipids used in this study were obtained from Avanti Polar Lipids, Inc. (Alabaster, AL) with ≥99% purity and used without further purification. 1,2-dioleoyl-*sn*-glycero-3-phosphocholine (DOPC) was supplied as a solution in chloroform. Monoolein (1-oleoyl-*rac*-glycerol) was purchased from Nu-Chek Prep, Inc. (purity ≥99%). Two types of oil, namely, squalene (SqE, 2,6,10,15,19,23-hexamethyl-2,6,10,14,18,22-tetracosahexaene) and squalane (SqA, 2,6,10,15,19,23-hexamethyltetracosane) were chosen for their ability to form essentially solvent-free droplet interface bilayers (DIBs).^30^ Perfluorooctanoic acid (PFOA, ammonium salt) and perfluorobutanesulfonic acid (PFBS, potassium salt) were purchased from Sigma-Aldrich at the highest available purity. All lipids were stored at −20 °C, while SqE was stored at 2–8 °C. Samples were freshly prepared immediately before use. For water permeability experiments, to prepare lipid-in-oil solutions, the chloroform solution of DOPC was first evaporated under inert gas to form a dried lipid film. This was followed by overnight vacuum drying. Subsequently, SqE was added to the dried film to achieve a final total lipid concentration of 5 mg/mL. PFOA or PFBS were added to the aqueous droplet phase. For differential scanning calorimetry (DSC) and attenuated total reflectance infrared (ATR-IR) spectroscopy, dried lipid films were rehydrated with aqueous PFOA or PFBS solutions to a total lipid concentration of ∼16 mg/mL. The aqueous lipid suspension was vortexed for 5 min to obtain multilamellar vesicles (MLVs), followed by 30 min of bath sonication. For Raman microspectroscopy, these MLVs underwent seven additional freeze–thaw cycles using liquid nitrogen. Interfacial tensiometry samples were prepared by dissolving monoolein powder in SqA (5 mg/mL), or DOPC film in SqE (5 mg/mL), followed by brief vortexing and bath sonication. All aqueous solutions were prepared using deionized water (18.2 MΩ·cm) purified by a Millipore Direct Q-3 system. Solution osmolality was measured using a VAPRO model 5600 vapor pressure osmometer immediately after preparation and prior to use.

### Differential Scanning Calorimetry

Thermal phase transition studies were conducted using a TA Q2000 Differential Scanning Calorimeter (DSC). The main phase transition behavior of the samples was analyzed using TA Universal Analysis software. The main phase transition temperature (*T*_m_) was determined as the temperature at the apex of the endothermic transition peak, while the phase transition enthalpy (Δ*H*) was calculated by integrating the area under the heat capacity curve. Approximately 15 μL of multilamellar vesicles (MLVs), prepared as described previously, were hermetically sealed in DSC pans. Samples were subjected to heating and cooling cycles at a rate of 5 °C/min, ranging from −40 to 0 °C, under a high-purity nitrogen atmosphere with a flow rate of 50 mL/min. To ensure reproducibility and assess potential hysteresis effects, each sample underwent three consecutive heating and cooling cycles. In all cases, consistent results were obtained across cycles. The reported values represent the average of measurements from three independently prepared samples and are expressed as mean ± standard deviation. Additionally, baseline subtraction and curve-fitting simulations were performed using OriginPro 10.1 software to deconvolute and fit into two components corresponding to higher and lower *T*_m_ regions.

### Attenuated Total Reflectance-Fourier Transform Infrared Spectroscopy (ATR-FTIR)

The ATR-FTIR spectral analysis was conducted using a Thermo Scientific Nicolet iS20 spectrometer equipped with a deuterated triglycine sulfate (DTGS) detector. Measurements were performed using a GladiATR single-reflection ATR accessory featuring a diamond crystal and temperature-controlled plate (Pike Technologies, USA). For each measurement, approximately 40 μL of MLVs, prepared as described in the experimental section, were applied to the diamond crystal surface. An ATR liquid retainer and volatiles cover accessory (Pike Technologies, USA) were used to contain the sample. Spectra were collected in the 400–4000 cm^–1^ range at 25 °C, with 200 scans accumulated and a spectral resolution of 4 cm^–1^. Background spectra using deionized water were recorded before each new sample and subtracted from the sample spectra. After each measurement, the diamond crystal, ATR liquid retainer, and volatiles cover were thoroughly cleaned with isopropanol and allowed to dry before the next sample. To ensure reproducibility, three independent sets of samples were prepared, with each sample scanned twice for consistency. ATR-FTIR data represent the average of all samples and are expressed as mean ± standard deviation. Data processing was performed using the OMNIC 9 (Thermo).

### Confocal Raman Microspectroscopy

Raman spectroscopic experiments were performed using an inverted confocal Raman microscope system (XploRA INV, Horiba) which consists of a Raman spectrometer directly coupled to an inverted microscope (Nikon Eclipse Ti–U). The Raman setup includes an internal laser operating at 532 nm (air-cooled solid-state laser) and a thermoelectrically cooled CCD detector. A 10× microscope objective was used for focusing a 532 nm wavelength laser beam, and for collecting Raman scattered light, subsequently dispersed with a grating of 1800 lines per millimeter. The glass coverslips (#1.5) used as substrates for deposition of lipid bilayer films were rinsed with ethanol and blown dry with N_2_ prior to use. A sample (10 to 20 μL) of a lipid or lipid mixture MLV suspension, immediately after generation by a freeze–thaw process (as described in sample preparation section), was spread on the surface of the cleaned coverslip and the aqueous solvent was allowed to evaporate in a closed homemade chamber on top of a heating plate at about 30 °C, to form a solid supported lipid bilayer on a hydrophilic surface. Three independent samples were prepared and each sample was scanned in three different regions and the average value is reported. All Raman spectra described herein are obtained at ambient room temperature, ca. 25 °C.

### Water Permeability Measurement Using Droplet Interface Bilayer (DIB)

Water permeability measurements were conducted using model membranes formed by the droplet interface bilayer (DIB) method. The detailed experimental setup and procedure have been described elsewhere.^31^ The setup consists of an inverted microscope (Nikon Eclipse Ti–S with halogen lamp) combined with two hydraulic micropipet manipulators (Narishige), all supported on a vibration-isolated workstation (Newport). A camera (Andor Zyla sCMOS) is directly attached to the microscope for real-time recording of the generated microdroplets and their size changes. Glass micropipets with tapered ends, controlled by the manipulators, were used to dispense aqueous microdroplets. Two osmotically unbalanced aqueous droplets, each approximately 100 μm in diameter, were created in a pool of squalene solvent containing lipids. One droplet contained pure water, while the other contained 0.1 M NaCl with a given concentration of PFOA or PFBS. All water permeability experiments were carried out at 30 °C using a custom-built temperature-controlled microchamber, which was thermostated via an external circulating water bath. The temperature of the microchamber containing lipid mixtures was measured using a thermocouple wire, with an accuracy of ±0.1 °C. Water permeability data represent an average of individual permeability runs (*n* ≥ 50), and standard deviation as error bars (mean ± standard deviation). The recorded videos and images were postanalyzed to measure the dimension of droplets and bilayer contact area, using custom built image analysis software. A detailed description of the method used to calculate water permeability can be found in the Supporting Information.

### Interfacial Tension and Contact Angle Measurement

Interfacial tension measurements at the oil–water interface were performed using a ramé-hart Advanced Goniometer/Tensiometer (model 590) in conjunction with DROPImage analysis software. These experiments were designed to study the surface adsorption of DOPC at SqE and monoolein at SqA-aqueous interfaces. Varying concentrations of PFOA or PFBS were introduced in the aqueous phase to assess their effects on interfacial properties. Typically, a 1 μL pendent drop of the aqueous phase was introduced into approximately 1 mL of the oil phase containing the lipid. For contact angle (θ) measurements, two iso-osmotic droplets were brought into proximity using micropipets and then allowed to make contact. The detailed methodology for contact angle measurement is provided in the Supporting Information.

## Results and Discussion

### Phase Transition Behavior of DOPC Membranes upon Exposure to PFOA and PFBS: DSC Studies

Figure 2 and Table 1 present the endothermic differential scanning calorimetry (DSC) thermograms and corresponding thermodynamic data for DOPC multilamellar vesicles (MLVs) in the presence of varying concentrations of PFOA and PFBS. The DSC thermogram of DOPC MLVs (Figure 2, control) reveals a well-defined endothermic transition at *T*_m_ of approximately −17 °C, with an associated enthalpy of about 9 kcal/mol. This data aligns with existing literature values (*T*_m_ = – 18.3 ± 3.6 °C, Δ*H* = 9.0 ± 2.8 kcal/mol).^32^ This transition represents the phase change from the lamellar gel phase L_β_ to the lamellar liquid-crystalline state L_α_. The relatively low *T*_m_ of DOPC indicates that, at room temperature, its membrane exists in a disordered, fluidic state—a characteristic typically associated with the presence of unsaturated acyl chains. Figure 2 and Table 1 illustrate that the phase transition of DOPC is similarly affected by both PFOA and PFBS, resulting in a concentration-dependent shift toward lower transition temperatures (*T*_m_) and an overall broadening of the transition. When PFOA is incorporated into the bilayer at a 100:1 mol ratio of DOPC to PFOA, the *T*_m_ decreases by approximately 0.3 °C compared to pure DOPC, accompanied by a slight reduction in enthalpy (Δ*H*) from 9.18 to 8.58 kcal/mol. The presence of PFBS at the same ratio results in a similar decrease in *T*_m_ by approximately 0.3 °C, but no significant change in Δ*H* (8.39 vs 8.40 kcal/mol). The changes in their thermograms become more pronounced at higher concentrations of PFOA and PFBS. At a 10:1 mol ratio of DOPC to either PFOA or PFBS, significant peak broadening is observed, accompanied by a low temperature shoulder (see Table 1 and Figure S1 in the Supporting Information). Note that the PFAS concentration in the 10:1 MLV suspension is 2 mM. The full width at half-maximum (fwhm) values for the peaks are 4.75 °C for PFOA and 4.41 °C for PFBS, respectively, at a DOPC/PFAS mole ratio of 10:1, compared to 2.27 °C for the case of pure DOPC. This phenomenon becomes more evident at higher concentrations. At a mole ratio of 6.7:1 DOPC/PFOA or PFBS, the peak splits into two components with distinct peak temperatures, as shown in Figure 2 and Table 1. Curve-fitting simulations were performed to deconvolute and fit the data into two components corresponding to lower and higher *T*_m_ regions (Figure S1 in the Supporting Information). The appearance of multiple peaks in DSC could plausibly be interpreted as phase separation or domain formation, with each peak corresponding to regions of different local PFAS composition. At 4:1 mol ratio of DOPC to PFOA or PFBS, a significant suppression in Δ*H* is observed, with enthalpy reduced from approximately 9 kcal/mol for pure DOPC to 1.5–2.0 kcal/mol for both PFOA and PFBS. Concurrently, the main phase transition shifts to lower temperatures by approximately 5 °C (from −16.7 to −21.7 °C) for PFOA and by about 2 °C (−17.1 to −19.1 °C) for PFBS. Note that the total area for all peaks was used for the enthalpy data for any thermogram having more than one peak.

**Figure 2:**
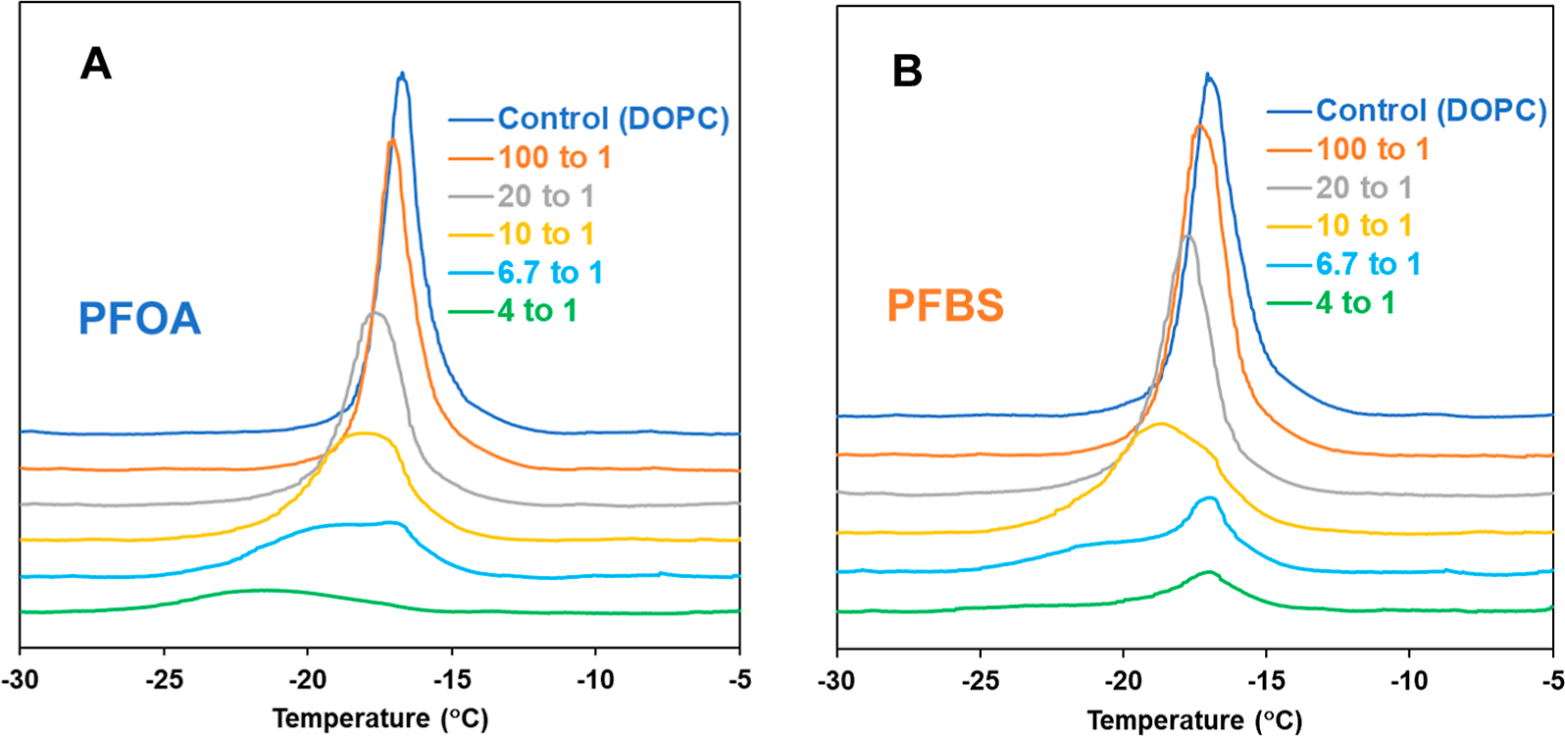
Endothermic calorimetric thermograms of DOPC MLVs containing (A) PFOA and (B) PFBS with varying concentrations.

**Table 1:** Thermodynamic Parameters (*T*_m_ and Δ*H*) for Main Phase Transition of MLVs of DOPC, with Varying PFOA and PFBS Concentrations

	PFOA		PFBS	
lipid/PFOA or PFBS (mol ratio)	peak(s), *T*_m_ (°C)	Δ*H* (kcal/mol)	peak(s), *T*_m_ (°C)	Δ*H* (kcal/mol)
1:0 (control)	–16.72 ± 0.22	9.18 ± 0.46	–17.06 ± 0.04	8.39 ± 0.04
100:1	–17.05 ± 0.17	8.58 ± 0.34	–17.36 ± 0.12	8.40 ± 0.02
20:1	–17.70 ± 0.05	7.09 ± 0.32	–17.75 ± 0.14	7.08 ± 0.13
10:1^a^	–17.26 ± 0.15 (31%); –18.77 ± 0.24 (69%)	6.03 ± 0.04	–17.26 ± 0.10 (23%); –19.08 ± 0.28 (77%)	5.46 ± 0.50
6.7:1^a^	–16.80 ± 0.21 (14%); –19.20 ± 0.72 (86%)	4.35 ± 0.15	–17.00 ± 0.53 (28%); –19.31 ± 0.83 (72%)	2.83 ± 0.11
4:1^a^	–18.40 ± 0.45 (14%); –21.70 ± 0.54 (86%)	1.56 ± 0.53	–17.06 ± 0.32 (49%); –19.07 ± 0.41 (51%)	1.97 ± 0.75

a At concentrations ≥10:1 (lipid/PFAS), curve-fitting simulations were performed using Origin software to deconvolute and fit into two components (Figure S1 in the Supporting Information). The enthalpy data represents the total area for all peaks. The relative percentage contribution to the total enthalpy is shown in parentheses.

Our DSC results demonstrate that both PFOA and PFBS interact with DOPC MLVs, influencing the bilayer assemblies and altering the bilayer’s thermotropic properties in a concentration-dependent manner. This interaction disrupts the lipid assemblies and the long-range order of the bilayer, as evidenced by the decrease in transition temperature and reduction in enthalpy. The observed shift to lower transition temperatures (*T*_m_), peak broadening, and reduction in enthalpy (Δ*H*) for DOPC membranes suggest reduced cooperativity, likely due to decreased van der Waals interactions among the hydrophobic chains when in the presence of PFOA and PFBS. At a lipid/PFAS ratio of 10:1 (and at higher PFAS content), the *T*_m_ peak splits into two, possibly indicating a transition to a biphasic lipid membrane. Increasing PFOA concentrations beyond this ratio further decreases both *T*_m_ peaks, whereas PFBS shows a plateau effect above 10:1. The continued decrease in *T*_m_ with rising PFOA concentration reflects its stronger interaction with the bilayer’s hydrophobic core compared to PFBS. This difference likely arises from PFOA’s greater membrane partitioning and deeper insertion. This is akin to prior quartz crystal microbalance studies using DMPC-supported bilayers, wherein PFOA suppresses the phase transition, while PFBS causes only a slight decrease in *T*_m_ at comparable concentrations.^33^ The putative phase separation observed at high concentrations of PFOA and PFBS aligns with earlier reports of domain formation in DOPC monolayers in the presence of PFOA and PFOS, as studied using Brewster Angle Microscopy.^34^ Our findings are qualitatively consistent with previous DSC studies involving phospholipids (albeit with saturated acyl chains), such as DMPC, DPPC and DSPC. For instance, it has been reported that both PFOA and PFOS, as well as PFBS induce a concentration-dependent decrease and broadening of the main phase transition temperature (*T*_m_), indicating disruption of lipid assemblies of DMPC, DPPC, and DSPC.^22,23,33^ It is important to note that our model membranes are based on DOPC (*T*_m_ = −17 °C), which is inherently fluidic at ambient temperature, in contrast to the saturated acyl chain PC of DMPC (*T*_m_ = 23 °C), DPPC (*T*_m_ = 41 °C) and DSPC (*T*_m_ = 55 °C).

### Structural Properties of DOPC Membranes upon Exposure to PFOA and PFBS: ATR-FTIR and Raman Spectroscopic Studies

In this section, we explore the effects of PFOA and PFBS on the structural properties of DOPC membranes using vibrational spectroscopic techniques, specifically ATR-FTIR and confocal Raman microspectroscopy.

#### ATR-FTIR Spectroscopic Studies

Phospholipid membranes generally exhibit three primary infrared-active stretching vibration regions: the lipid acyl chain (CH_2_, around 2850 and 2920 cm^–1^), the interfacial carbonyl group (C=O, around 1730 cm^–1^), and the phosphate headgroup (PO_2_^–^, around 1000–1240 cm^–1^). These regions are crucial for detecting structural and organizational changes in lipid bilayers upon interaction with bioactive molecules.^35–37^ Shifts in wavenumber and changes in bandwidth within these IR bands provide insights into the structural and dynamic modifications occurring within the lipid bilayer, offering a molecular-level understanding of interactions with membrane-active molecules. Figure 3A,C present ATR-FTIR spectra in the CH_2_ region (2820–2960 cm^–1^) for DOPC membranes in the presence of varying concentrations of PFOA and PFBS, respectively, at 25 °C. The spectra are normalized using the respective CH_2_ antisymmetric stretching vibration band at around 2920 cm^–1^ and vertically shifted. The tabulated wavenumbers in the CH_2_ region are provided in Table S1 (Supporting Information). An expanded view of the CH_2_ antisymmetric stretch (ν_as_) region (2910–2940 cm^–1^) is shown in Figure 3B,D to highlight peak shifts. Minimal shifts were observed in the symmetric stretching (ν_s_ CH_2_) part of the spectra (∼2853 cm^–1^). As illustrated in Figure 3B, our IR band analysis reveals noticeable wavenumber blue shifts (∼2 cm^–1^) in the CH_2_ antisymmetric stretching region (ν_as_), from 2923.1 ± 1.0 cm^–1^ (pure DOPC) to 2925.0 ± 1.0 cm^–1^ (4:1 DOPC/PFOA mole ratio). The blue shift in Ν_as_ CH_2_ suggests an increase in gauche conformers and reduced acyl chain order, indicating a more disordered state upon exposure of DOPC membranes to PFOA.^35^ A similar blue shift is observed in the presence of PFBS, albeit to a slightly lesser extent (∼1 cm^–1^), from 2923.1 ± 1.0 cm^–1^ to 2924.0 ± 1.0 cm^–1^ (Figure 3D). Additionally, changes in the bandwidth of the CH_2_ stretching modes reflect variations in acyl chain mobility, providing insights into membrane dynamics. Our spectra show an increase in the overall bandwidth of CH_2_ antisymmetric stretching vibration modes (Ν_as_ CH_2_) from 24.1 cm^–1^ in pure DOPC to 28.0 cm^–1^ at a 10 to 1 mol ratio of DOPC to PFOA. A similar, though less pronounced, band broadening was observed for PFBS exposure (from 24.1 cm^–1^ for pure DOPC to 25.9 cm^–1^ at a 10 to 1 mol ratio of DOPC to PFBS). This indicates increased acyl chain dynamics at high PFOA and PFBS concentrations, signifying membrane perturbation. Our findings are qualitatively consistent with previous studies on DMPC bilayers, which reported shifts in the Ν_as_ CH_2_ band toward higher frequencies (∼1–2 cm^–1^) and bandwidth broadening in the presence of PFOA or PFOS.^20^

**Figure 3:**
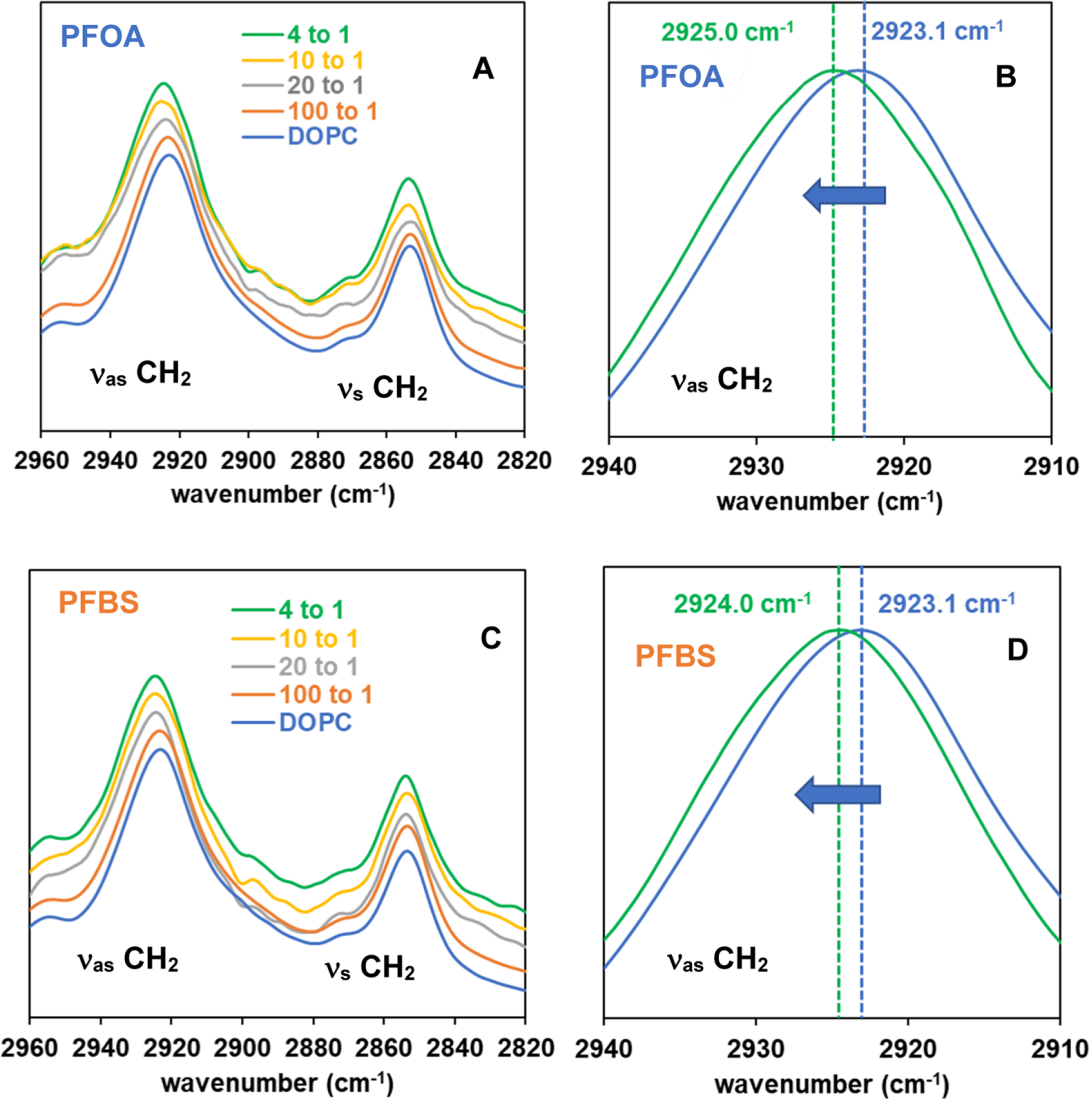
Representative ATR-IR spectra in the stretching vibration of the acyl chain CH_2_ groups of DOPC membranes, with varying mol ratios of (A) PFOA and (C) PFBS at 25 °C, with the expanded regions of interest of ν_as_ CH_2_ (B,D) highlighting the change at the highest concentration of PFAS. The vertical dotted lines in (B,D) indicate the wavenumber shift in the absence (control DOPC, blue) and presence of (B) PFOA and (D) PFBS (4 to 1 mol ratio of DOPC to PFOA or PFBS, green).

The phosphate headgroup region, characterized by the PO_2_^–^ symmetric and antisymmetric stretching vibration bands around 1000–1240 cm^–1^, provides insights into hydration and hydrogen bonding at the surfaces of hydrated phospholipid assemblies.^38^ However, in our system, the phosphate headgroup region overlaps with C–F vibration bands, complicating precise peak analysis. In addition, while the characteristics of the C=O stretching vibration region can provide information about the hydrogen bonding around the glycerol backbones of DOPC, the broad band around ∼1738 cm^–1^ in this region was not clearly resolvable in our spectra, limiting our ability to definitively interpret molecular interactions and structural changes in this region.

Our findings align with previous studies using IR reflection–absorption spectroscopy,^20^ which have suggested that PFOA and PFOS strongly interact with zwitterionic phospholipids such as DMPC, leading to an increased gauche conformation in the alkyl chains of DMPC. Similarly, studies from vibrational sum frequency generation spectroscopy has shown that perfluoroheptanoic acid perturbs the ordering of DPPC alkyl chains at lipid monolayers on the water surface, leading to less organized interfacial water associated with the phosphate.^21^ In summary, our findings on the characteristic changes in vibrational modes within the CH_2_ region provide evidence that both PFOA and PFBS interact with DOPC membranes and influence the conformation of the lipid assembly in a concentration-dependent manner.

#### Raman Spectroscopic Studies

We utilized confocal Raman microspectroscopy of supported lipid bilayers to investigate structural changes in DOPC membranes upon exposure to PFOA and PFBS molecules. Figure 4 presents the ambient temperature Raman spectra in the frequency range of 600–3600 cm^–1^ for DOPC supported bilayers containing varying concentrations of PFOA. All spectra were normalized to the intensity of the peak at ∼2850 cm^–1^ (the most intense peak) to facilitate comparison. Corresponding spectra for DOPC exposed to PFBS are provided in the Supporting Information (Figure S2).

**Figure 4:**
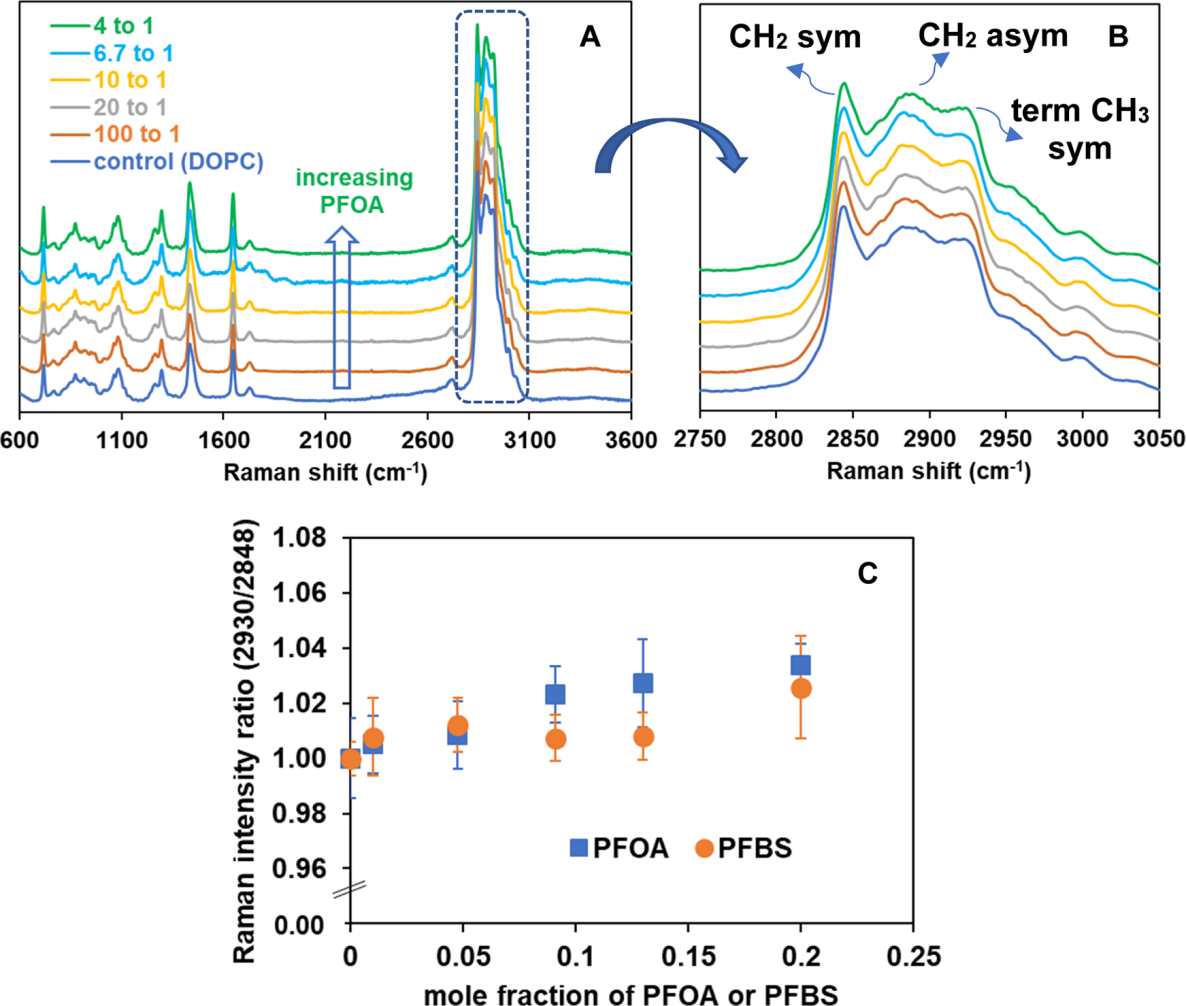
(A) Representative Raman spectra of DOPC at various concentrations of PFOA at ambient temperature, (B) the expanded region of stretching CH band. Spectra are normalized to the intensity ∼2850 cm^–1^ (the most intense peak) for comparison, and vertically shifted for clarity, and (C) relative Raman intensity ratios of [C–H_term_ (2930)/C–H_sym_ (2848)] as a function of concentrations of PFOA and PFBS in DOPC. Each data point represents average and standard deviation for three different regions across the sample from at least 3 independently prepared samples.

Figure 4B highlights the C–H stretching region, spanning from 2750 to 3050 cm^–1^, which is notable for its intense Raman scattering in phospholipid molecules and the association of peaks in this region with hydrocarbon chain order in lipid membranes. Within this region, characteristic peaks for DOPC are observed: the methylene C–H symmetric stretching mode appears around 2850 cm^–1^, the antisymmetric methylene C–H stretching mode near 2890 cm^–1^, and the symmetric stretching mode of the terminal methyl C–H group at approximately 2930 cm^–1^. These peaks, and especially their relative intensity ratios, serve as valuable markers for assessing membrane structural characteristics. Specifically, the intensity ratio between the terminal C–H and symmetric C–H stretching modes is sensitive to intermolecular chain coupling.^39,40^ Figure 4C demonstrates that, as the concentration of PFOA in DOPC increases, there is a small but gradual rise in the relative peak intensity ratio of [C–H_terminal_ (2930)/C–H_symmetric_ (2848)]. In comparison to PFOA, an even lesser change is observed for PFBS, except at the highest concentrations. The corresponding Raman intensity ratios of [C–H_terminal_ (2930)/C–H_symmetric_ (2848)] is tabulated in Table S2. As the hydrocarbon chains decouple and intermolecular interactions diminish, the terminal methyl groups gain enhanced rotational and vibrational freedom. This results in an increased intensity ratio of the symmetric stretching mode of the terminal methyl group to that of the methylene group. An increase in this [C–H_terminal_/C–H_symmetric_] intensity ratio typically indicates greater freedom of motion, rotational disorder, and increased lipid molar volume in the hydrocarbon chain region. Consequently, the observed increase in ratios with higher molecular concentrations suggests that molecules interacting with the DOPC lipid bilayer influence intermolecular chain coupling, progressively inducing a disordering effect on the DOPC lipid bilayers, with a greater extent of such change for PFOA but similarly disruptive effect for PFBS at high concentrations.

### Membrane Barrier Properties upon Exposure to PFOA and PFBS: Water Permeability Studies

In this section, we investigate how PFOA or PFBS may affect the integrity of membranes with respect to their permeability, since disruptive structural changes can compromise the barrier function of the membrane, making it more porous to small molecules. The transport of water across biological membranes is crucial for cellular physiology and maintaining homeostasis. In our prior studies, we designed a method using droplet interface bilayers (DIBs) to examine how different membrane compositions affect water permeability in model membrane systems.^41,42^ The DIB functions as a model membrane system. It forms when aqueous microdroplets, each surrounded by a lipid monolayer in oil, come into contact, creating a bilayer region at their interface.^43^ The interdroplet contact zone in DIB closely mimics the double-leaflet lipid bilayer found in cellular membranes (see Figure 5A) and provides a versatile platform for biomimetic modeling of cellular membranes, offering unique opportunities to explore chemistry at the nanoscale within lipid bilayers.^42,44^ These prior studies, utilizing DIB-based water permeability assessments, have revealed that water transport through a single unsupported bilayer is sensitive to the physical state of model membranes.^42^ This sensitivity allows us to detect subtle changes in membrane composition and structure,^31,45^ as well as the effects of exogenous bioactive molecules.^46–48^

**Figure 5:**
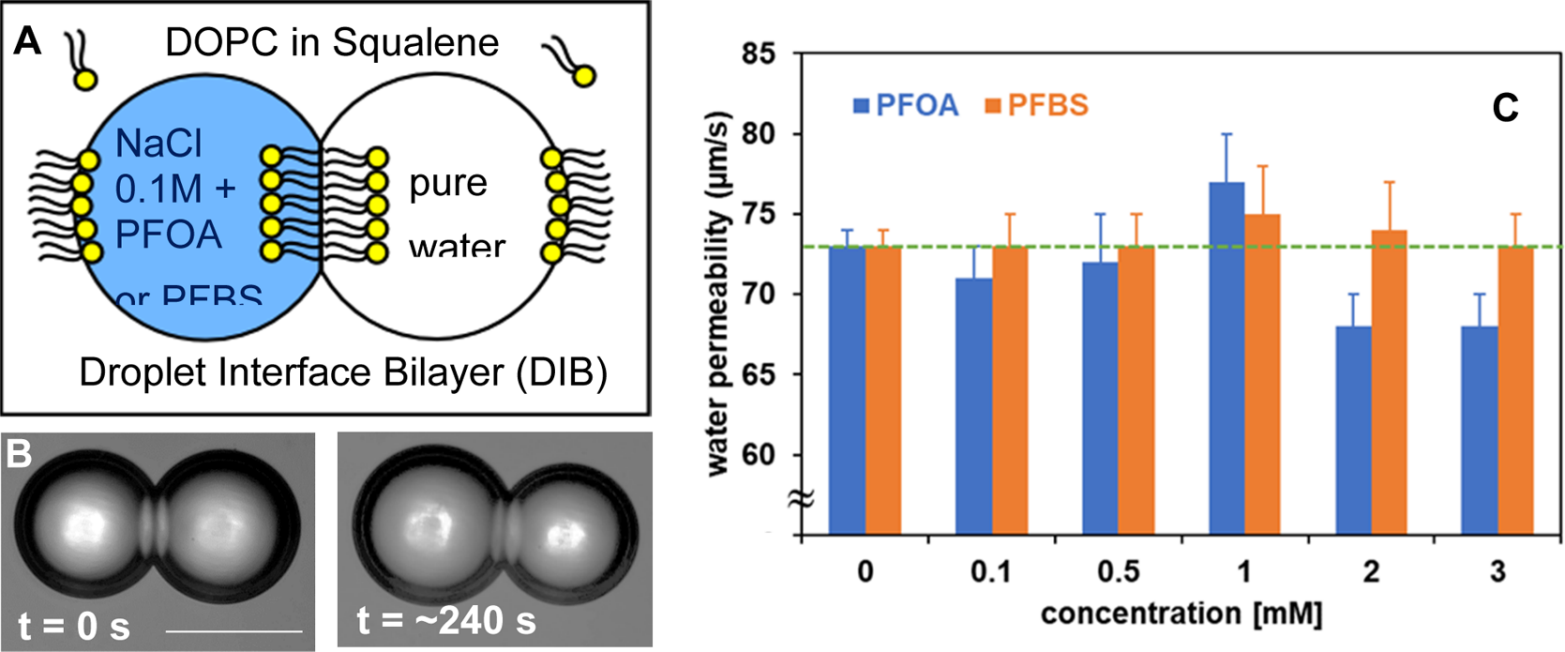
(A) General schematic of the DIB-based osmotic water permeability experiment, (B) typical micrographs showing droplets (each droplet is about 100 μm in diameter at its initial size) undergoing osmotic swelling and shrinkage upon transport of water molecules across DOPC lipid membranes, which provides the bases for the osmotic water permeability calculation (a blue arrow indicates the direction of water transport), and (C) osmotic water permeability coefficients (μm/s) of lipid bilayer formed from DOPC at 30 °C with varying concentrations of PFOA or PFBS. The dotted horizontal green line shows a control value. Each data point represents an average of individual permeability runs (*n* ≥ 50), and standard deviation as error bars. The scale bar on the videomicrograph represents 100 μm.

Figure 5A shows a schematic description of the DIB-based water permeability measurement system, designed to detect the effect of PFOA or PFBS. A pair of osmotically unbalanced aqueous droplets are used, one containing pure water and the other containing 0.1 M NaCl with varying concentrations of PFOA or PFBS (up to 3 mM). When an osmotic pressure imbalance exists between two adhering aqueous droplets in a DIB, water transport occurs through the droplet bilayer, resulting in a noticeable change in droplet diameter (Figure 5B). The changes in droplet volume over time are measured optically through microscopic observation. The recorded videos capturing these changes are analyzed afterward using custom-built image analysis software to measure the relevant parameters needed to determine the water permeability coefficient (see additional details in the Supporting Information). Note that for a droplet with a nominal diameter of 100 μm containing PFOA or PFBS, where the droplet surface is coated with DOPC (area per lipid, approximately 70 Å^2^), the ratio of lipid molecules to PFOA or PFBS molecules can be estimated as being approximately 1.42 (at 0.1 mM) and 0.14 (at 1 mM), respectively. This ratio is comparable in magnitude to what we have studied using DSC and vibrational spectroscopy described in previous sections.

Figure 5C presents the osmotic water permeability coefficients (*P*_f_) of DOPC-based membranes at 30 °C, illustrating how these coefficients vary with different concentrations of PFOA or PFBS. The corresponding permeability coefficients are provided in Table S3 (Supporting Information). In Figure 5A, PFOA or PFBS is introduced at specific concentrations in the leftmost droplet containing 0.1 M NaCl. The results shown in Figure 5C demonstrate that the *P*_f_ for water transport changes as the concentration of PFOA increases (blue column in Figure 5C). Compared to the control DIB formed from pure DOPC (dotted horizontal green line), there are insignificant changes below 1 mM concentrations of PFOA and PFBS, although slightly decreasing trends in *P*_f_ for PFOA is observed, while no changes are seen in PFBS. At 1 mM, both PFOA and PFBS shows moderate increase in water permeability, from 73 ± 1 μm/s (control) to 77 ± 3 μm/s (PFOA), and to 75 ± 3 μm/s (PFBS). However, above 1 mM concentrations of PFOA, a decline in water permeability became apparent, to 68 ± 2 μm/s. We note that this is the same PFOA concentration regime at which DSC begins to exhibit splitting in the phase transition peak (see Table 1), indicating a possible association between the two phenomena. Further increase in concentration from 2 mM to 3 mM showed that the reduction in *P*_f_ values for PFOA was maintained. In this regime, PFBS engenders little or no reduction in *P*_f_ (74 ± 3 μm/s), relative to control (73 ± 1 μm/s). Overall, the presence of PFOA show more distinct concentration dependent changes in *P*_f_ values, compared to that of PFBS where the changes are insignificant at the concentration range we studied.

In general, the permeability of water through lipid bilayers depends on several structural and physical properties of the individual lipids and the bilayers they form. These properties include bilayer thickness, the molecular area occupied by each lipid, and the overall membrane fluidity.^49–51^ The fluidity or rigidity of bilayers is usually linked to the packing density of lipids,^52^ and it is anticipated that water permeability is affected by how tightly lipids are packed within the bilayer region. At 1 mM, our data showing an increase in water permeability for both PFAS molecules tested may reflect the structural disruption of the DOPC membrane environment in the presence of these molecules and the compromised intermolecular interactions between lipid headgroups, which can contribute to an enhanced fluidity. However, at higher concentrations (2–3 mM), the apparent reduction in transbilayer water permeability suggest a potential rigidity in the bilayer. A decrease in membrane fluidity upon exposure to PFAS has been reported by others, including previous studies using neutron spin–echo techniques. These studies have shown that PFOA integrates into the membrane in an alternating distribution with lipid molecules, restricting the rotation of lipid acyl chains and exerting a stiffening effect on the DMPC bilayer.^18^ Additionally, molecular dynamics (MD) simulations indicate that a range of PFAS molecules (PFOA, PFOS, and perfluorononanoic acid) spontaneously penetrate the lipid bilayer. Once embedded, these PFAS molecules have a condensing effect on the bilayer, similar to the effect of cholesterol.^17^ Calcein leakage experiments demonstrated that various perfluorinated compounds mitigated nanoparticle-induced leakage in DOPC liposomes, implying an improvement in liposomal membrane stability.^19^ MD simulations show that PFOS-containing DPPC bilayers have a smaller area per molecule and thicker membrane compared to pure DPPC bilayers, indicating a condensing effect on the model membrane.^53^ However, NMR studies on DMPC bilayers reveal concentration-dependent effects of PFOA. At low concentrations, PFOA addition decreases the lateral diffusion of DMPC molecules, suggesting a membrane condensing effect. However, at higher PFOA concentrations (exceeding 5 mol %), the lateral diffusion coefficient increases, indicating enhanced bilayer fluidity.^54^ It has also been reported that PFAS can cause condensation of lipid membrane based on the compressibility test, while yielding a more fluid and compressible monolayer.^34^ These observations suggest that PFAS exposure induces heterogeneous effects on membrane structure, potentially leading to phase separation where some regions exhibit increased lipid packing (condensation), while others show enhanced fluidity. This structural heterogeneity could explain the observed biphasic behavior in water permeability across the membrane, in which low PFAS concentrations (1 mM) increase permeability slightly whereas higher value decrease *P*_f_. The interaction mechanisms of PFOA and PFBS are undoubtedly complex and can involve multiple forces, including electrostatic interactions, van der Waals forces, and hydrogen bonding. These interactions collectively influence the packing characteristics of DOPC lipid membranes, which can be observed in the changes in water permeability values, as demonstrated here. Moreover, the differing impacts on water permeability observed with PFOA and PFBS highlight how sensitive these molecules are to variations in their molecular structures. Recent findings^55^ provide mechanistic support for our observation of increased water permeability at low concentrations. This study demonstrates that PFOA disrupts the structure of DPPC vesicles—even at submicromolar concentrations—by impairing chain–chain interactions and lipid packing, and thereby increasing the accessibility of the hydrophobic interior to secondary solutes. These results would reinforce our conclusion that increased water permeability in the low concentration regime arises from PFOA-induced alterations in membrane structure and dynamics.

### Interfacial Activity Behaviors at the Water–Oil Interface upon Exposure of PFOA and PFBS: Bilayer Tension

In this section, we employ a combination of interfacial tension measurements and interdroplet contact angle analysis to quantify the interfacial adsorption behavior and the membrane perturbation capability of PFOA and PFBS at water–oil interfaces and water–water (bilayer) interfaces in lipid systems. Numerous PFAS molecules exhibit surfactant-like properties, demonstrating a propensity to accumulate at the boundaries between different liquid phases.^56^ One of the key physicochemical characteristics of biological membranes is the tension that exists across their bilayer structure, commonly referred to as membrane tension or bilayer tension. This property is intimately linked to the membrane’s rigidity and overall stability.^57^ The surface energy manifested as bilayer tension in biological membranes plays a significant role in cellular processes and is associated with various cellular activities, particularly influencing the efficiency of membrane fusion events and the functionality of membrane-associated proteins.^58^ The values of membrane tension in a DIB can be readily determined through knowledge of the relevant interdroplet contact angle (θ) formed by the droplets at their interface and the liquid–liquid interfacial tension (γ_m_) for each monolayer, as measured by a pendant drop tensiometer.^44,59^ This approach provides a robust method for quantifying the interfacial behavior of PFAS in lipid systems, offering valuable insights into their potential interactions with biological membranes.

In the present tension study, in addition to DOPC, we employed a bilayer system based on the single-chain lipid, monoolein. This lipid rapidly forms long-lasting droplet interface bilayers and has been extensively used for studying bilayer tension and its changes, both in black lipid membranes and droplet interface bilayers.^60,61^ We determined the bilayer tension (γ_b_) by combining the monolayer interfacial tension (γ_m_) with the interdroplet contact angle (θ) measured between two adherent droplets, as described in the experimental section (and Figure S3). The relationship is expressed by eq 1

1 $$\gamma_{b}=2\gamma_{m}\;\cos\;\theta$$

Tables 2 and 3 show the effects of varying concentrations of PFOA and PFBS on monolayer tension, contact angle, and bilayer tension of monoolein and DOPC membranes, respectively.

**Table 2:** Interfacial Activities of PFOA and PFBS at the Water/Monoolein-Squalane Interface at 25 °C^a^

	monolayer tension, γ_m_ (mN/m)		contact angle, θ (degrees)		bilayer tension, γ_b_= 2γ_m_ cos θ (mN/m)	
PFOA or PFBS Conc. (mM)	PFOA	PFBS	PFOA	PFBS	PFOA	PFBS
0	1.249 ± 0.019	1.170 ± 0.032	28.56 ± 0.20	30.57 ± 0.21	2.19 ± 0.06	2.01 ± 0.10
0.004	1.219 ± 0.009	1.198 ± 0.023	30.05 ± 0.23	30.49 ± 0.16	2.11 ± 0.02	2.06 ± 0.07
0.04	1.148 ± 0.011	1.202 ± 0.038	30.83 ± 0.37	30.68 ± 0.13	1.97 ± 0.02	2.07 ± 0.12
0.4	0.924 ± 0.010	1.180 ± 0.050	31.43 ± 0.23	30.73 ± 0.17	1.58 ± 0.03	2.03 ± 0.17
0.8	0.766 ± 0.008	1.141 ± 0.038	33.58 ± 0.39	30.99 ± 0.18	1.28 ± 0.02	1.96 ± 0.12
1.2	0.579 ± 0.003	1.112 ± 0.050	36.79 ± 0.36	31.10 ± 0.22	0.93 ± 0.01	1.90 ± 0.16
2.0	0.299 ± 0.006	1.076 ± 0.067	36.73 ± 0.42	31.72 ± 0.17	0.48 ± 0.01	1.83 ± 0.22

a Each data point represents average and standard deviation (SD) for *n* = ≥ 10 trials.

Our results demonstrate that increasing PFOA concentration in the aqueous phase enhances its adsorption at the bilayer interface, as evidenced by a reduction in bilayer tension from 2.19 mN/m in the absence of PFOA to 0.48 mN/m in the presence of 2 mM PFOA, for the monoolein bilayer interface (Table 2). In contrast, PFBS at the same concentration induced a much smaller decrease in bilayer tension (from ∼2 to 1.8 mN/m) for the monoolein bilayer interface (Table 2). Similar qualitative trends are observed for the DOPC bilayer interface (Table 3). A greater reduction in bilayer tension is observed for PFOA (from 1.63 to 0.55 mN/m at 0.4 mM PFOA), compared to PFBS (from 1.63 to 1.02 mN/m), as shown in Table 3.

**Table 3:** Interfacial Activities of PFOA and PFBS at the Water/DOPC–Squalene Interface at 25 °C^a^

	monolayer tension, γ_m_ (mN/m)		contact angle, θ (degrees)^b^		bilayer tension, γ_b_= 2γ_m_ cos θ (mN/m)	
PFOA or PFBS Conc. (mM)	PFOA	PFBS	PFOA	PFBS	PFOA	PFBS
0	0.963 ± 0.173	0.963 ± 0.173	32.41 ± 1.20	32.41 ± 1.20	1.63 ± 0.43	1.63 ± 0.43
0.004	0.758 ± 0.125	0.993 ± 0.210	36.31 ± 2.03	36.00 ± 0.78	1.22 ± 0.31	1.61 ± 0.65
0.04	0.467 ± 0.122	1.040 ± 0.175	39.13 ± 2.44	37.77 ± 1.97	0.72 ± 0.33	1.64 ± 0.47
0.4	0.380 ± 0.132	0.653 ± 0.135	43.14 ± 1.23	38.33 ± 0.32	0.55 ± 0.36	1.02 ± 0.41

a Each data point represents average and standard deviation (SD) for *n* = 5 trials.

b Images of contact angle of a DIB pair are shown in Figure S4 (Supporting Information).

Our findings reveal distinct patterns of interfacial behaviors for PFOA and PFBS, providing valuable insights into their interactions with biological membranes. PFOA consistently demonstrates a more pronounced impact on lipid bilayer tensions compared to PFBS. Increased PFOA concentrations lead to a significant reduction in bilayer tension, likely due to the intercalation of PFOA molecules into the hydrophobic region of the model membranes. This intercalation alters the packing and organization of lipid molecules. In contrast, PFBS exhibits a less pronounced effect on bilayer tension, highlighting the different interfacial properties of these two PFAS. We attribute this disparity primarily, although not exclusively, to the difference in hydrocarbon chain length between PFOA (*n* = 8) and PFBS (*n* = 4). The reported experimental logarithmic membrane/water partition coefficients (log *K*_mem/w_) for PFOA and PFBS are 3.52 ± 0.08 and 2.86 ± 0.06, respectively.^62^ The longer perfluorinated chain of PFOA facilitates stronger hydrophobic interactions with the lipid bilayer, leading to more efficient intercalation into, and greater interaction with, the membrane structure. Further support for the differential behavior of PFOA and PFBS at membrane interfaces is provided by recent work by Sobolewski et al.^63^ Their Langmuir study demonstrated that PFOA exhibits significantly greater surface activity and a stronger propensity to aggregate at hydrophobic interfaces, than does PFBS. These findings are consistent with our observations of PFOA’s more pronounced effects on membrane modifying properties.

## Conclusions

This study provides a comprehensive evaluation of the concentration-dependent impacts of long-chain PFOA and short-chain PFBS on the biophysical properties of the DOPC model membrane. Our findings demonstrate that both PFAS compounds perturb zwitterionic DOPC membranes at bilayer level, with effects that vary according to concentration and molecular structure. Water permeability data for PFOA reveal a concentration-dependent biphasic characteristics, suggesting a heterogeneous impact on membrane organization. This complexity is further supported by the observed phase separation in DSC studies, which indicate the formation of heterogeneous membrane structures with regions of varying lipid packing and fluidity. Vibrational spectroscopy experiments further suggest that PFAS exposure increases the flexibility and movement of DOPC molecules, resulting in a higher number of gauche conformations and enhanced overall membrane fluidity. Notably, PFOA and PFBS exhibited distinct impacts on membrane interfacial properties indicating the sensitivity of PFAS-membrane interactions to differences in molecular structure. The relatively high membrane/water partition coefficients of PFOA compared to PFBS highlight the significant role of the hydrophobic effects in long-chain versus short-chain PFAS interactions. However, as these two PFAS differ in both chain length and headgroup, the observed differences reflect the combined influence of these structural features. Importantly, PFBS, despite its shorter chain length, induced substantial structural modifications in model membranes at high concentrations, suggesting that replacing long-chain PFAS with shorter alternatives may not be as environmentally benign as anticipated. These findings enhance our understanding of PFAS–membrane interactions and provide a foundation for elucidating how PFAS exposure may impact cellular processes associated with membrane dynamics. Although our experimental PFAS concentrations (0.004 mM–5 mM) generally exceed typical human serum levels (10 to 10^4^ ng/mL, or 0.02 μM to 0.02 mM), it is important to recognize that PFAS distribution in the body is highly uneven, with some tissues accumulating much higher concentrations than blood.^7,11,64^ Measurements of mean composition may underestimate localized accumulation, and, as observed with drug–membrane interactions, local concentrations can exceed tissue averages due to membrane heterogeneity.^65^ Thus, PFAS may also accumulate in specific microenvironments at elevated levels.

Recent consensus highlights the critical role of membrane lipid organization in regulating cellular function. The formation of distinct lipid domains within the plasma membrane can profoundly influence the localization, structure, and activity of membrane-associated proteins.^66^ For example, changes in domain composition or fluidity can modulate the function of ion channels, receptors, and enzymes by altering their conformational dynamics or assembly within the membrane.^66^ Furthermore, increased membrane permeability and altered domain structure may impact membrane fusion processes, which are essential for vesicular trafficking, neurotransmitter release, and other cellular events. Recent studies have shown that small molecules such as serotonin can promote vesicle association and fusion by modifying lipid bilayer properties, emphasizing the importance of the lipid environment in fusion dynamics.^67^ Any disruption of lipid packing and domain organization by PFAS may thus have consequences for cellular signaling, transport, and homeostasis, as many membrane proteins are sensitive to their lipid nanoenvironment. It is plausible to envision that PFAS-induced changes in membrane order and permeability could similarly affect the energetics and efficiency of membrane fusion, potentially altering normal cellular trafficking and communication.

In summary, our research contributes to a deeper understanding of how PFOA and PFBS modify cell membranes at the molecular level. These insights are crucial, as disturbances in the lipid bilayer can affect protein activities and functions. Our findings illuminate the complex nature of PFAS-membrane interactions and underscore the importance of considering molecular structure when predicting the environmental and health impacts of these persistent chemicals.

## Supplementary Material



## Notes

DSC thermograms with curve-fitting simulations; ATR-IR wavenumber with varying concentrations of PFOA or PFBS in the region of CH_2_ stretching vibrational bands; Raman spectra of DOPC at various concentrations of PFBS; Raman intensity ratios of [C–H_term_ (2930)/C–H_sym_ (2848)] of DOPC in the presence of PFOA or PFBS; Analysis of water permeability data; Water permeability coefficient for DOPC as a function of PFOA or PFBS concentrations; contact angle measurement and images (PDF)
