# Supplementary material for: Biophysical Consequences for Exposure of Model Cell Membranes to Perfluoroalkyl Substances
Source: J Phys Chem B. 2025 Jul 29;129(31):7951–63. doi: 10.1021/acs.jpcb.5c02472 (PMC12337092; doi:10.1021/acs.jpcb.5c02472)
Supplement: Supplementary file 1 [file jp5c02472_si_001.pdf]

Supporting Information

Biophysical Consequences for Exposure of Model Cell Membranes to Perfluoroalkyl Substances

*Joseph Fosella[a], Jasmin Ceja-Vega[a], Amani Rabadi[a], Micaela Panella[a], Jessica Said[a], Wilber Perla[a], Christopher Poust[a], Mary Herrera[a], and Sunghee Lee[a]**

a Department of Chemistry and Biochemistry, Iona University, 715 North Avenue, New Rochelle, NY 10801, United States

* E-mail: SLee@iona.edu

Figure S1. Deconvolution of DSC thermogram Page S2

Table S1. Wavenumber with varying concentrations of PFOA or PFBS at 25°C in the region of CH_2 stretching vibration bands. Page S3

Figure S2. Representative Raman spectra of DOPC at various concentrations of PFBS at ambient temperature. Page S4

Table S2. Raman intensity ratios of [$C{-}H_{term}$ (2930)/$C{-}H_{sym}$ (2848)] of DOPC Page S4

Text. Analysis of Water Permeability Data Page S5

Table S3. Water permeability coefficient at 30 °C for DOPC membrane, as a function of PFOA or PFBS concentratons. Page S6

Text. Contact Angle Measurement Page S7

Figure S3. A schematic of two adherent droplets illustrates the contact angle Page S7

Figure S4. Contact angle of a DIB pair in the presence of DOPC in squalene Page S7

Phase transition behavior of DOPC membranes upon exposure of PFOA and PFBS: Deconvolution of DSC Thermograms

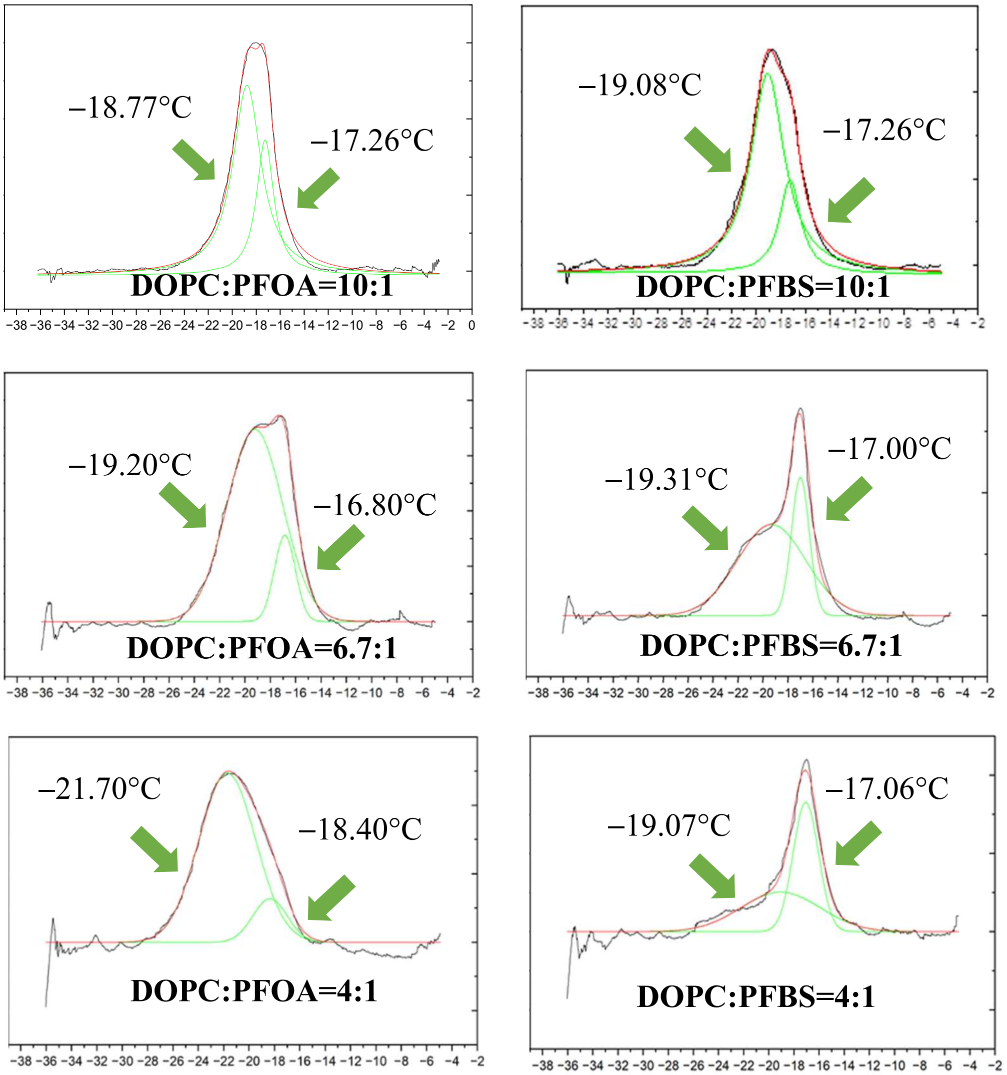

Figure S1. Curve-fitting simulations were performed using OriginPro 10.1 software to deconvolute and fit into two components.

ATR-IR Spectroscopy

Table S1. Wavenumber with varying concentrations of PFOA or PFBS at 25°C in the region of CH_2 stretching vibration bands.

Concentration of PFOA or PFBS	Wavenumber (cm^{-1}), ν_{as} CH_2		Wavenumber (cm^{-1}), ν_s CH_2	
	DOPC with PFOA	DOPC with PFBS	DOPC with PFOA	DOPC with PFBS
control	2923.1 ± 1.0	2923.1 ± 1.0	2853.2 ± 1.0	2853.2 ± 1.0
100 : 1	2923.6 ± 1.0	2923.6 ± 1.0	2853.2 ± 1.0	2853.2 ± 1.0
20 : 1	2924.0 ± 1.0	2924.0 ± 1.0	2853.6 ± 1.0	2853.2 ± 1.0
10 : 1	2925.0 ± 1.0	2924.0 ± 1.0	2854.1 ± 1.0	2853.6 ± 1.0
4 : 1	2925.0 ± 1.0	2924.0 ± 1.0	2854.1 ± 1.0	2853.6 ± 1.0

Raman Spectroscopy

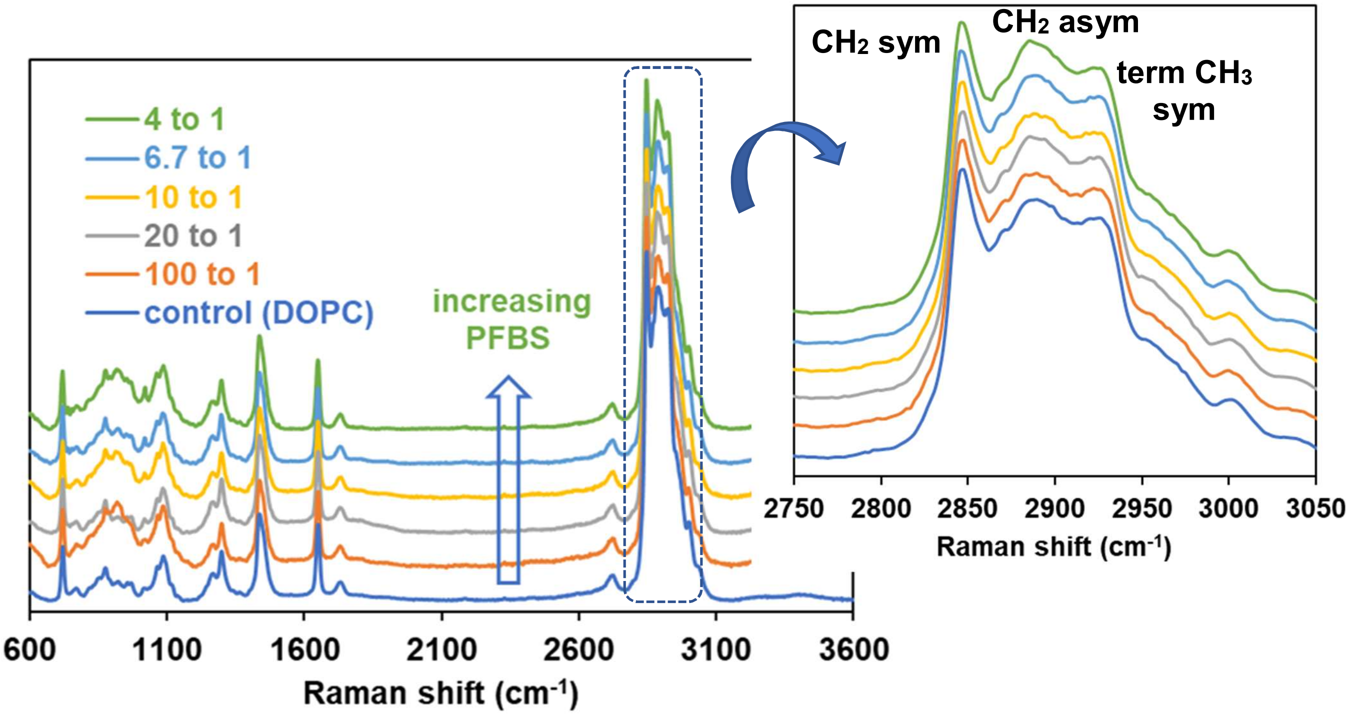

Figure S2. Representative Raman spectra of DOPC at various concentrations of PFBS at ambient temperature, and the expanded region of stretching CH band. Spectra are normalized to the intensity ~2850 cm^{-1} (the most intense peak) for comparison and vertically shifted for clarity.

Table S2. Raman intensity ratios of [C–H_{term} (2930)/C–H_{sym} (2848)] of DOPC at ambient temperature.

DOPC:PFAS (mol:mol)	Raman intensity ratio (I=2930/2848)	
	PFOA	PFBS
1:0	0.833 ± 0.015	0.832 ± 0.012
100:1	0.837 ± 0.010	0.839 ± 0.014
20:1	0.840 ± 0.012	0.842 ± 0.010
10:1	0.852 ± 0.010	0.838 ± 0.008
6.7:1	0.856 ± 0.016	0.839 ± 0.009
4:1	0.861 ± 0.008	0.854 ± 0.019

Analysis of Water Permeability Data

When two osmotically unbalanced microdroplets adhere at a bilayer, the osmotic gradient propels water transport through the droplet bilayer (as indicated by the arrow in Figure 6 in the main article), leading to a noticeable change in droplet diameter. Any electrolyte flux is expected to be negligible compared to that of water, as ion permeation is typically almost eight orders of magnitude slower than that of water. The corresponding changes in droplet volume over time (dV/dt) is measured optically by microscopic observation; and the behavior of the system follows the expression of equation (1) based on Fick's Law:

$$\frac{dV(t)}{dt} = -P_f A(t) v_w \Delta C(t) \qquad (1)$$

where A is the geometric bilayer area, ν_w is the molar volume of water (18 mL/mol), $\Delta C(t)$ is the time-dependent osmolality gradient between two droplets, and P_f is the bilayer permeability coefficient of water. The volume change with time (dV/dt) is related to the bilayer permeability coefficient of water, P_f, as expressed in the Equation (1). When the bilayer contact area is constant, the time evolution of the swelling droplet can be obtained from the following equation derived from the integration of eqn. 1, with the following simplifying assumption: since one of the droplets (the shrinking droplet) contains no osmotic agent, its concentration does not change with time:[1, 2]

$$\left(\frac{V}{V_o}\right)^2 = \left(\frac{2P_f A v_w C_o}{V_o}\right)t + 1 \qquad (2)$$

Using the measured values for: initial size of the osmotic (swelling) droplet; bilayer contact area (A); and initial osmolarity of the osmotic droplet (C_o), then the coefficient P_f for bilayer water permeability may be derived from eqn. 2 from the slope of the curve obtained by plotting $(V/V_o)^2$ as a function of time. All data points presented in this paper are an average ($n \geq 50$) of individual permeability runs, each of which took place over a time course (~5 min) for osmotic water movement across the droplet bilayer, during which time the droplet contact area (A) remains constant. The recorded videos and images were post-analyzed to measure the dimension of droplets and contact area using custom built image analysis software. All droplet pairs had substantially the same initial size relative to each other, in the diameter range of 100 ± 5 µm diameter.

---

[1] Lopez, M.; Evangelista, S. E.; Morales, M.; Lee, S. Enthalpic effects of chain length and unsaturation on water permeability across droplet bilayers of homologous monoglycerides. *Langmuir* 2017, 33 (4), 900-912.
[2] Thiam, A. R.; Bremond, N.; Bibette, J. From stability to permeability of adhesive emulsion bilayers. *Langmuir* 2012, 28 (15), 6291-6298.

Table S3. Water permeability coefficient at 30 °C for DOPC membrane, as a function of PFOA or PFBS concentratons. Each data point represents an average of individual permeability runs (n ≥ 50), and standard deviation as error bars.

PFOA or PFBS (mM)	Water permeability coefficient (μm/s)	
	DOPC with PFOA	DOPC with PFBS
0	73 ± 1	73 ± 1
0.1	71 ± 2	73 ± 3
0.5	72 ± 3	73 ± 3
1.0	77 ± 3	75 ± 3
2.0	68 ± 2	74 ± 3
3.0	68 ± 2	73 ± 2

Contact Angle Measurement

The schematic of a droplet pair shown in Figure S4 were formed in a chamber containing lipid in SqE. From the microscopic video images of the two adherent droplets, the contact angle can be measured by considering the geometry of the contacting spheres (as given in eqn. 3) based on geometrical parameters shown in Figure S3,

$$2\theta = sin^{-1}\left(\frac{r}{R_1}\right) + sin^{-1}\left(\frac{r}{R_2}\right) \qquad (3)$$

where, R_1 and R_2 are the radii of the respective two droplets and r is the radius of the contact zone between the droplets.

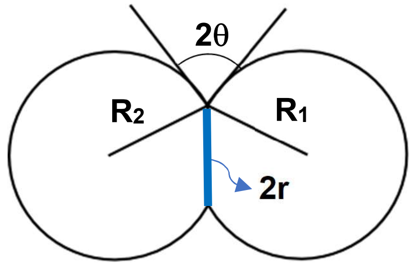

Figure S3. A schematic of two adherent droplets illustrates the contact angle (θ), which is determined by equation 3.

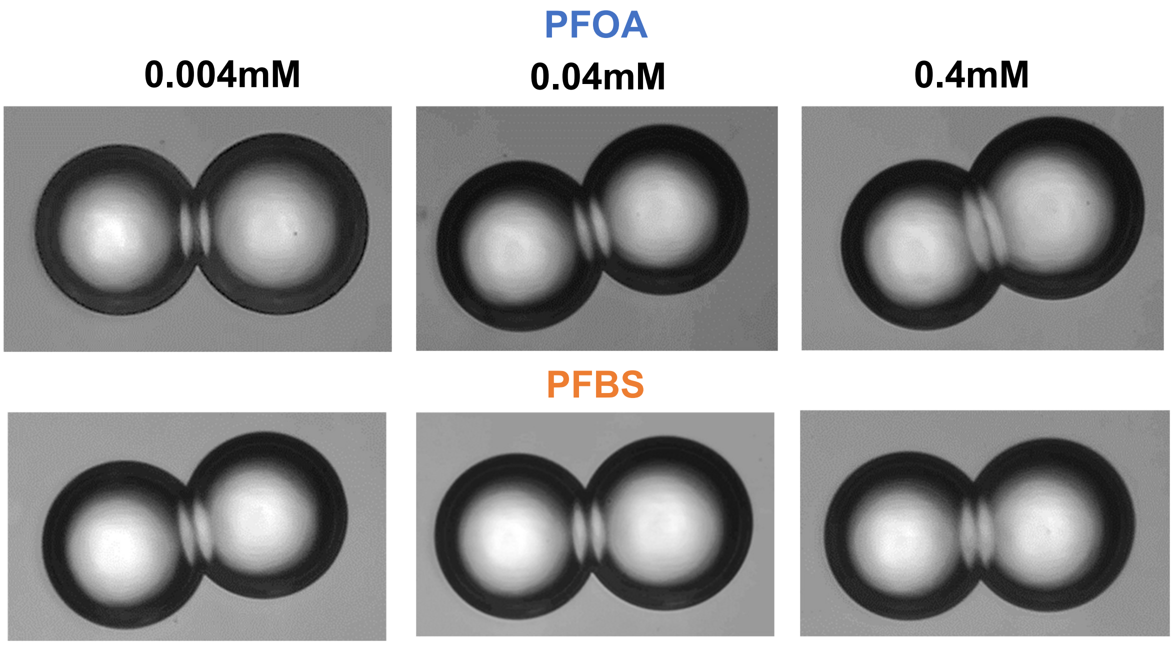

Figure S4. Contact angle of a DIB pair in the presence of DOPC in SqE (5mg/mL). An aqueous droplet pair contains a defined PFOA or PFBS concentrations.
